# Virtual network sampling method using LinkedIn

**DOI:** 10.1016/j.mex.2021.101393

**Published:** 2021-05-21

**Authors:** Arkadiusz Kozłowski, Adam Kaliszewski, Janusz Dąbrowski, Hanna Klimek

**Affiliations:** a Department of Statistics, Faculty of Management, University of Gdańsk, ul. Armii Krajowej 101, Sopot 81-824, Poland; b Institute of Maritime Transport and Seaborne Trade, Faculty of Economics, University of Gdańsk, Armii Krajowej 119/121, Sopot 81-824, Poland

**Keywords:** Hard-to-reach populations, Nonprobability sampling, Global surveys, Snowball sampling, Online surveys

## Abstract

Surveying people via the Internet presents both opportunities and challenges. The opportunities include quick access to respondents all over the world enabling fast realization of surveys at low costs. The challenges include a lack of control over both the sampling process and research implementation, as well as low response rates. This article describes a method of obtaining an appropriate sample of people for survey research utilising the power of the Internet, while simultaneously allowing the researcher to minimise risk thanks to enhanced control over the sampling process and the implementation of the survey. The proposed method could be used mainly in economic and social surveys; it allows researchers to reach selected groups of respondents and to conduct surveys on a global scale.

• The proposed sampling method uses LinkedIn's network structure to quickly reach a dispersed population
• Creating a list of units belonging to the study population resembles the snowball method, though the units are selected for the sample by the researcher and not indicated by the respondents.

## Graphical abstract

**Figure:**
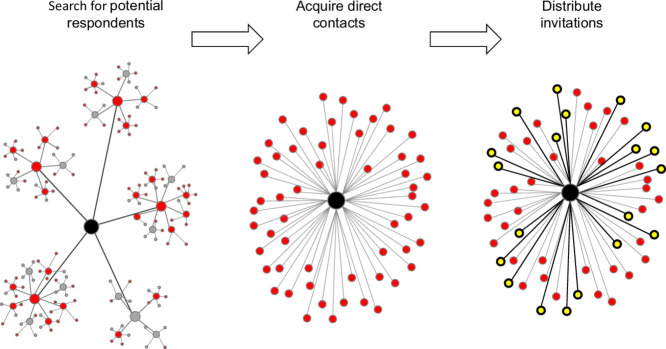

Specifications table

**Table:** 

Subject Area:	Economics and Finance
More specific subject area	Sampling methods for surveys of people
Method name:	LinkedIn network sampling
Name and reference of original method:	Virtual snowball sampling Baltar, F. and Brunet I. (2012) *Social research 2.0: virtual snowball sampling method using Facebook*, “Internet Research”, Vol. 22, No. 1, pp. 57-74, https://doi.org/10.1108/10662241211199960
Resource availability:	not applicable

## Method details

In economic research, the global perspective is increasingly important. This is due to the significant globalization of many areas of economic life, e.g. maritime trade, tourism, and the production of electronics or the production of cars. When conducting scientific research on groups related to a given industry and scattered around the world, the problem of reaching these people and drawing the appropriate sample arises.

One of the possible solutions to this problem is the selection of respondents via LinkedIn, which is the world's largest platform for employees from various industries. LinkedIn, like other large Internet platforms, has a network structure, i.e., each user is ``connected'' to a certain group of people, each with their own group of connections, and so on, very quickly creating a vast network of potential respondents.

The sampling method proposed resembles the snowball method. Specifically, its development in the form of RDS - Respondent Driven Sampling [6], and in the online version the virtual snowball sampling method [3]. In contrast with these methods, however, the initial respondents do not recommend more people for the study, something which is usually a source of bias. Hence, the snowball analogy only applies to the building of a list of population units, which occurs without the active participation of these units.

The sampling procedure consists of three steps:

1. Building a list of potential respondents belonging to the study population
2. Acquiring respondents from the created list as direct contacts of the researcher
3. Distributing invitations to participate in the study

Ad.1. Using the LinkedIn Sales Navigator (Professional) tool, a subscription-based service with a free introductory period, search for individuals belonging to the survey population. The search may be based on a list of previously prepared organisations as the respondent's workplaces. There are many criteria available to adjust the scope of the search to find individuals specified by the needs of the research in question, including location, industry, and position. The search takes place within the network of connections of the account owner (researcher), i.e., in the first, second or third degree of connection, or in virtual groups of professionals. Therefore, the success of this stage depends on having a diverse initial network of the researcher's first contacts and the time allowed for subsequent iterations.

Ad. 2. Contact invitations should be sent to the persons from the created list, so that these persons appear as the first contact of the researcher (links cannot be included in invitations). The researcher can use LinkedIn Helper (a paid tool, with a free introductory period) to automatically send a large number of personalized invitations.

The key to the success of this stage is the inviting person - the researcher (this may be, for example, a person working in the same industry as the invitees) and the justification for the invitation to contact. In case of additional questions from invitees, make sure to reserve time and resources for correspondence, practically on around the clock basis for a global audience due to time zones.

Ad. 3. After the second step, the researcher should have a diverse panel of units belonging to the study population. The last element of the procedure involves sending invitations to participate in the survey (very simple polls can be carried out within LinkedIn, more advanced questionnaires must be carried out on a different platform). At this stage, the researcher may decide on a quota selection so that the sample has an appropriate structure with respect to the characteristics specified by the researcher.

The success of this stage depends on the appropriate wording of the invitation to take part in the survey - incentives can be used here, e.g. access to the survey report, previous publications or other offers that will be attractive from the respondent's point of view.

To streamline the acquisition process, a simple programming can be applied to indicate {First Name} of a target person, who is a mutual connection with {First Name of 1^st^ degree connection} or alternatively highlight that both the researcher and a target person belong to the same {Interest Group}. A limitation of this approach is with some Asian names, as they use initials or family names only, which may indicate to the recipient that the invitation is computer generated.

Advantages of LinkedIn network sampling:

• Low costs (financial costs include subscriptions to LinkedIn Sales Navigator and LinkedIn Helper)
• The use of the LinkedIn network structure that allows you to effectively reach a specific group of respondents
• Personalized communication between the researcher and the respondent, increasing the motivation to participate in the study
• No geographic barriers
• The utilization of looser connections within the network of friends - unlike, for example, RDS, the respondent does not participate in building the sample, but the researcher himself selects the next respondent, exploring the network of connections between people, so that it corresponds to the objectives of the study, regardless which persons an already surveyed respondent would recommend.

Disadvantages of LinkedIn network sampling:

• The study population is limited to those who use LinkedIn
• Potentially high refusal rate when: a) requested to connect, b) requested to participate in the survey. In both cases, a bias may arise that depends on the researcher – the LinkedIn account owner, and the objectives of the study.

## Method validation

LinkedIn network sampling was used in two studies on maritime trade. Both studies focused on the competitiveness factors of container ports.

In the first study [7,8], the study population consisted of shipping lines senior managers and directors. The primary search criterion in the first stage was a list of the world's largest shipping lines, according to Alphaliner's TOP 100 [1]. The survey lasted 2.5 months, a significant part of which was devoted to direct correspondence with potential respondents. At the second stage, over 1,000 people were acquired as first contacts of one of the researchers (the fact that the inviting researcher was also an employee of this industry, which could be verified, had a significant impact on the willingness to connect). The invitation to participate in the study was sent to all new contacts; the survey itself was carried out on the LimeSurvey platform. The incentive to take part in the study was access to the authors' previous publications. A total of 210 persons attempted the survey, with 120 full, useable responses recorded. The final sampling cost per respondent with complete answers was $ 0.91.

The second study [9,10] concerned employees of forwarding companies. The initial query to LinkedIn Sales Navigator was based on the ControlPay Freight Audit 2018 list of top 50 ocean freight forwarders [4]. The respondents list was limited by seniority to manager and above, as well as by function to operations, sales, and business development. The implementation and final sampling efficiency were similar to the first study. This time, 164 people began the study, and 113 completed the questionnaire.

In both cases, it was possible to obtain samples whose structures - in terms of geographic differentiation and the size of the enterprises in which the respondents worked - were approximately the same as the known distributions of these characteristics in the population. It should be emphasized that the success of the described sampling method in these cases was significantly influenced by the background of the main researcher, who was also an employee of the industry in question, and the time and energy devoted to personal correspondence with potential respondents.

## Conclusion

The virtual network sampling method making use of LinkedIn allows one to draw a representative sample of professionals from around the world to conduct an online survey. The method utilizes existing networks of connections between potential respondents (similar to the snowball method or respondent driven sampling), but it does not need any active involvement of network members in the process. In addition, the selection process uses publicly available information about the surveyed individuals (information provided by LinkedIn users), which increases the credibility of the sample and permits control over its composition. The cost of obtaining a list of potential respondents and conducting a survey is small compared to other methods designed for a global scale. The most important factor when implementing the method is for the main researcher to be themselves part of the studied population, e.g. by working in the same industry.

## Additional information

Statistical research, in which the subject of the study is a large population of people, is most often carried out on a sample of that population. The selection of a representative sample of people is crucial for the credibility of the research results. The desired way of selecting a sample is probability sampling (see e.g. [11]), because the probabilities of individual units getting into the sample are then known, which allows the use of the entire spectrum of statistical inference methods. However, probability sampling entails the necessity to have a sampling frame, i.e. a list of all population units. In practice, the sampling frame is often impossible to obtain or build, or is prohibitively expensive. In such situations, nonprobability sample selection methods come to the rescue (see e.g. [2]). Though these methods do not allow for the use of rigorous statistical inference, (a certain mathematical inference model is still applicable, see Elliot and Valliant, [5]), they do enable the selection of a sample more cheaply and quickly, while controlling the representativeness of a sample.

The Internet provides many opportunities in terms of sample selection, as well as research implementation. Much of the research, both commercial and scientific, in the past carried out using the face-to-face method, is now carried out online. Research via the Internet has its drawbacks, of course, but their undeniable advantage is the lack of geographical barriers, which is particularly important in research conducted on a global scale. The herein proposed procedure for selecting respondents is one possible way of reaching a survey population scattered all over the world.

## Declaration of Competing Interests

The authors declare that they have no known competing financial interests or personal relationships that could have appeared to influence the work reported in this paper.
